# Effect of asking questions and providing knowledge on attitudes toward organic foods among Japanese consumers

**DOI:** 10.3389/fpsyg.2023.1274446

**Published:** 2023-12-27

**Authors:** Shuma Iwatani, Hidehito Honda, Yurina Otaki, Kazuhiro Ueda

**Affiliations:** ^1^Graduate School of Arts and Sciences, The University of Tokyo, Tokyo, Japan; ^2^Faculty of Psychology, Otemon Gakuin University, Osaka, Japan; ^3^Graduate School of Social Sciences, Hitotsubashi University, Tokyo, Japan

**Keywords:** self-assessed knowledge, illusion of explanatory depth, intervention, organic food, willingness to pay

## Abstract

Some people overestimate the benefits of certain kinds of foods, such as organic foods, while others underestimate it. Previous studies have found that reducing people’s self-assessed knowledge successfully moderated these extreme attitudes. In this study, we investigated interventions to reduce people’s self-assessed knowledge and to moderate attitude extremity. We examined extreme attitudes toward organic foods and investigated the effects of implementing two intervention methods to moderate their attitude: (1) providing knowledge on organic food after asking them some questions and (2) simply providing them with knowledge. We conducted a two-factor mixed-design experiment with 653 college-educated Japanese women. In the first condition, before knowledge provision, participants were asked to answer questions about organic foods and were then informed of the correct answer and whether their answer was correct (Q&A Intervention). This step was based on an intervention conducted in a previous study to reduce their self-assessed factual knowledge. In the second condition, participants were simply provided with knowledge without being asked to answer any questions (Simple Intervention). The results showed that both intervention methods, on average, decreased the participants’ self-assessed knowledge and attitude extremity. Therefore, simply providing knowledge may be effective in reducing their self-assessed factual knowledge and moderating their extreme attitudes toward organic foods.

## 1 Introduction

### 1.1 People’s attitudes toward healthy foods

People have different attitudes toward food. For example, people with less knowledge tend to consume smaller amounts of vegetables and fruits (Hill et al., 2020; for a review see Shaikh et al., 2008). Such people are more likely to face problems such as cardiovascular disease, as these foods are linked to a lower incidence of cardiovascular disease (Slavin and Lloyd, 2012). Stanaway et al. (2022) meta-analyzed studies from various countries including those in Europe, Asia, and North America, and demonstrated that consuming smaller amounts of vegetables was associated with an increased risk of ischemic stroke, esophageal cancer, and type 2 diabetes. However, having extremely positive attitudes toward specific foods is not recommended either. Exclusively eating vegetables and fruits can also be detrimental to one’s health, as consuming in a variety of foods, such as dairy products, meat, and carbohydrates is recommended in the United States (Dixon et al., 2001) and Korea (Lim, 2018). Indeed, there was a negative relationship between physical frailty and varieties of foods people ingested among older adults in Japan (Kiuchi et al., 2021).

As the above example reflects, some people’s attitudes toward specific foods are extreme (Fernbach et al., 2019). When people are informed that a food is superior in one aspect (e.g., “organic food has a positive effect on environmental sustainability”), they may mistakenly believe that the food is also superior in another aspect (e.g., “organic food has a positive effect on human health,” although this claim has not been sufficiently proven; Vigar et al., 2019). This cognitive bias is known as the food halo effect (Roe et al., 1999), which can polarize people’s attitudes. In particular, people with extremely positive attitudes toward specific foods often falsely believe that they have certain effects on health and disease; this phenomenon is called food faddism (Jarvis, 1983). For example, people who followed the Zen Macrobiotic Diet consumed natural foods to cure diseases, such as heart disease and cancer, although there was no scientific evidence to support this (McBean and Speckmann, 1974).

In this study, we examine the effects of interventions to moderate people’s extremely positive or negative attitudes toward foods. We focus on organic foods as “foods perceived as healthy” and suggest an intervention to moderate attitudes.

### 1.2 Different attitudes toward organic foods

Previous studies, which surveyed consumers in Germany, Spain, and Japan, demonstrated some of them had positive attitudes toward organic foods. Bauer et al. (2013) conducted in-depth interviews with German consumers and demonstrated that one of their main reasons to buy organic foods was their perceived high nutrition. They also conducted an online experiment and demonstrated that food items labeled as organic were perceived more healthy and environmentally friendly. According to Vega-Zamora et al. (2014), consumers in Spain perceived organic foods as healthy or natural. Among the Japanese, the most common reason for starting to consume organic foods is disease prevention (Ministry of Agriculture, Forestry and Fisheries, 2019), implying that Japanese have positive attitudes toward organic foods in terms of their health. Indeed, some studies have demonstrated that those who regularly consume organic foods have low risks of obesity, type 2 diabetes, postmenopausal breast cancer, and lymphoma (Kesse-Guyot et al., 2022). However, the long-term effect of organic foods on human health is unclear because longitudinal intervention studies have not been conducted. Consequently, research is inconclusive on the positive effects of organic foods on human health. Therefore, consumers who perceive organic foods as healthy are considered to have extremely positive attitudes toward organic foods.

In contrast to the studies that demonstrated positive attitudes toward organic foods, other studies demonstrated the existence of negative attitudes. For example, more than half of Japanese consumers do not eat organic foods even once a month (Ministry of Agriculture, Forestry and Fisheries, 2018). Aschemann-Witzel and Zielke (2017)’s review claimed that one of the reasons consumers do not buy organic foods is the high price, but their perceived price was outdated and higher than actual. In other aspects, some Swiss consumers believe that even cultivating organic foods pollutes the environment and avoid purchasing them (Hansmann et al., 2020). According to Northen (2011)’s review, people’s trust in organic foods can decrease when they perceive organic foods as a form of *greenwashing*, defined as “the intersection of two firm behaviors: poor environmental performance and positive communication about environmental performance” (Delmas and Burbano, 2011, p. 65). However, their perceptions of organic foods being not good for environment needs to be corrected because organic farming methods have more environmental benefits than conventional farming (Gomiero et al., 2011). Considering these incorrect beliefs, consumers who have incorrect high-price image toward organic foods or those who do not trust the positive effect of the foods on the environment may have extremely negative attitudes toward them.

In this study, we applied the findings obtained in the cognitive science literature about the illusion of explanatory depth (i.e., overconfidence in their knowledge; Rozenblit and Keil, 2002) to moderate people’s attitudes toward organic foods. This study implemented two kinds of interventions (Simple Intervention and Q&A Intervention) for the purpose of (1) reducing people’s self-assessed factual knowledge and (2) moderating their attitudes. Self-assessed factual knowledge refers to an individual’s estimation of how much they know about certain facts, such as the criteria for food to be certified as organic. People who overestimate their knowledge tend to have an incorrect or extreme attitude (Fernbach et al., 2013, 2019). Therefore, reducing people’s self-assessed factual knowledge is important for moderating attitude extremity. We implemented two intervention methods for this: (a) Simple Intervention, which simply provides participants with factual knowledge, and (b) Q&A Intervention, which provides participants with factual knowledge after asking them some questions.

In the following section, we first describe the relationship between people’s self-assessed knowledge and their attitudes (subsection 1.3.1.). Then, we introduce the method for reducing their self-assessed knowledge in subsection 1.3.2. and describe the purpose of this study in subsection 1.4, applying this method to moderate people’s extreme attitudes toward organic foods. Finally, in subsection 1.5, we will describe our hypotheses.

### 1.3 Literature review

#### 1.3.1 An antecedent factor of wrong judgments: overestimation of self-assessed knowledge

People sometimes make inaccurate judgments, such as believing fake news to be true (Pennycook and Rand, 2020). Previous studies have demonstrated that misjudgments, such as believing fake news, are associated with self-assessed knowledge (e.g., Pennycook and Rand, 2020). Self-assessed knowledge refers to people’s “estimates of how much they know or have learned about a particular domain” (Sitzmann et al., 2010, p. 169). People tend to overestimate their knowledge, and this overestimated self-assessed knowledge affects their attitudes. In their study about fake news, Pennycook and Rand (2020) demonstrated that people who overestimate their knowledge are more likely to judge fake news as accurate.

The overestimation of self-assessed knowledge also affects attitude extremity (Fernbach et al., 2013). People overestimating their understanding of policies (e.g., the impact of imposing unilateral sanctions on Iran for its nuclear program) tended to have more extreme political attitudes (Fernbach et al., 2013). It is also demonstrated that overconfident people (e.g., those who believe their estimation of the current unemployment rate is accurate, though their estimation is wrong) have more extreme attitudes (Ortoleva and Snowberg, 2015).

#### 1.3.2 Interventions to reduce self-assessed knowledge

The phenomenon that people overestimate their self-assessed knowledge is called the *illusion of explanatory depth*; it occurs when “people feel they understand complex phenomena with far greater precision, coherence, and depth than they really do” (Rozenblit and Keil, 2002, p. 521). According to Rozenblit and Keil (2002), people have such an illusion because they have few opportunities to provide explanations about things, thereby not realizing the actual amount of knowledge they possess.

To prevent people’s wrong or extreme attitudes toward foods, investigating strategies that reduce the overestimation of self-assessed knowledge is essential. Rozenblit and Keil (2002) provided their participants with an opportunity to offer detailed explanations regarding a mechanism and succeeded in reducing their self-assessed knowledge. In their study 1, participants were asked to write a step-by-step causal explanation of the mechanism behind a flush toilet. After the intervention, participants’ self-assessed knowledge about this mechanism was lower than that before the intervention. This shows that individuals’ self-assessed knowledge can be lowered through an intervention that allows them to provide in-depth explanations of a phenomenon, making them aware of their lack of knowledge.

Fernbach et al. (2013) applied Rozenblit and Keil’s (2002) intervention in reducing people’s self-assessed knowledge to moderate people’s extreme political attitudes. In their study, participants were asked to (1) report their political attitudes (for or against some policies, such as establishing a cap-and-trade system for carbon emissions), (2) judge their self-assessed knowledge regarding political issues, (3) explain some policies, such as the impact of instituting a cap-and-trade system for carbon emissions, and (4) describe their political attitude and self-assessed knowledge again. The researchers tried to reduce the participants’ self-assessed knowledge by asking them to explain the policies [procedure (3)]. The intervention successfully reduced the participants’ self-assessed knowledge, moderating the extremity of their political attitudes.

### 1.4 Purpose and originality

As described above, overestimating self-assessed knowledge is a factor in people’s attitude extremity. Therefore, in this study, we tried to reduce people’s self-assessed knowledge and moderate people’s extreme attitudes.

We focused on self-assessed factual knowledge rather than self-assessed explanatory knowledge. Factual knowledge refers to how well people know about certain facts, such as the criteria for being certified as organic foods, while explanatory knowledge refers to “knowledge that involves complex causal patterns” (Rozenblit and Keil, 2002, p. 522), such as the mechanism how some foods improve or worsen people’s health. Providing factual knowledge is a common intervention to modify people’s attitudes toward foods. For example, the “5 a day campaign” tries to provide factual knowledge that eating at least five daily servings of fruit and vegetables is beneficial for health (Heimendinger et al., 1996) rather than providing people with explanatory knowledge such as the biological mechanism of how eating fruits and vegetables promotes health. Therefore, focusing on factual knowledge is practically important.

We tried to reduce their self-assessed factual knowledge (i.e., the definition of organic foods) and moderate their attitudes toward certain foods. Although the overestimation of factual knowledge is weaker than that of explanatory knowledge, people overestimate their factual knowledge as well (Rozenblit and Keil, 2002), which may lead to extreme attitudes (Fernbach et al., 2019).

Our study has at least two original aspects. First, we extended Rozenblit and Keil’s (2002) intervention into a consumer research study. We attempted to moderate people’s attitudes toward foods through Rozenblit and Keil’s intervention to reduce self-assessed knowledge. Second, we extended Fernbach et al.’s (2013) study by investigating whether intervention to reduce people’s self-assessed *factual* knowledge could change their attitudes. This focus differs from that of Fernbach et al. (2013), which mainly involved explanatory knowledge.

### 1.5 Hypothesis development

First, we focus on intervention to reduce self-assessed knowledge. We implemented two intervention methods: Simple Intervention and Q&A Intervention. Simple Intervention refers to the intervention to simply provide participants with factual knowledge. We assumed that Simple Intervention did not reduce people’s self-assessed knowledge. Imagine a situation where people have a wrong belief and are provided with knowledge that contradicts it. If people accept the provided knowledge, they will understand that their previous belief is wrong, which will then reduce their self-assessed knowledge. However, people do not always accept information that contradicts their beliefs (Sunstein et al., 2016). Therefore, we estimated that people would not accept simply-provided knowledge, and thus, Simple Intervention would not reduce people’s self-assessed knowledge.

In contrast to Simple Intervention, Q&A Intervention refers to an intervention where participants are asked to answer some questions before they are provided with knowledge (Rozenblit and Keil, 2002). Rozenblit and Keil (2002) demonstrated that people’s self-assessed knowledge after Q&A Intervention was lower than that before the intervention. Therefore, Q&A Intervention could help people realize the actual amount of knowledge they possess, which would reduce their self-assessed factual knowledge. Based on their study, we examined the following hypothesis:

*H1*: The difference (reduction) in self-assessed knowledge between before and after the Q&A Intervention is greater than the difference in self-assessed knowledge between before and after the Simple Intervention.

As mentioned in section 1.3.1., a large amount of self-assessed knowledge can strengthen people’s attitude extremity (Fernbach et al., 2013; Ortoleva and Snowberg, 2015). Based on this finding, we assumed that Q&A Intervention would moderate participants’ attitude extremity because it could reduce participants’ self-assessed knowledge (as hypothesized in H1). Meanwhile, we assumed that Simple Intervention would not moderate participants’ attitude extremity because Simple Intervention would not reduce participants’ self-assessed knowledge (as hypothesized in H1). Based on this assumption, we examined the following hypothesis:

*H2*: The difference (reduction) in attitude extremity between before and after the Q&A Intervention is greater than the difference in attitude extremity between before and after the Simple Intervention.

In this study, we conducted Simple Intervention as well as Q&A Intervention. We conducted Simple Intervention because Q&A Intervention not only reduces people’s self-assessed knowledge but also increases their actual knowledge. Without Simple Intervention, even when Q&A Intervention successfully moderates people’s attitudes, we cannot conclude whether it is a reduction in self-assessed knowledge or an increase in actual knowledge that moderates people’s attitudes. By comparing these two types of interventions, we aimed to examine the effect of reducing people’s self-assessed factual knowledge on their extreme attitudes while controlling the effect of increasing their amount of knowledge.

## 2 Materials and methods

**Figure 1:**
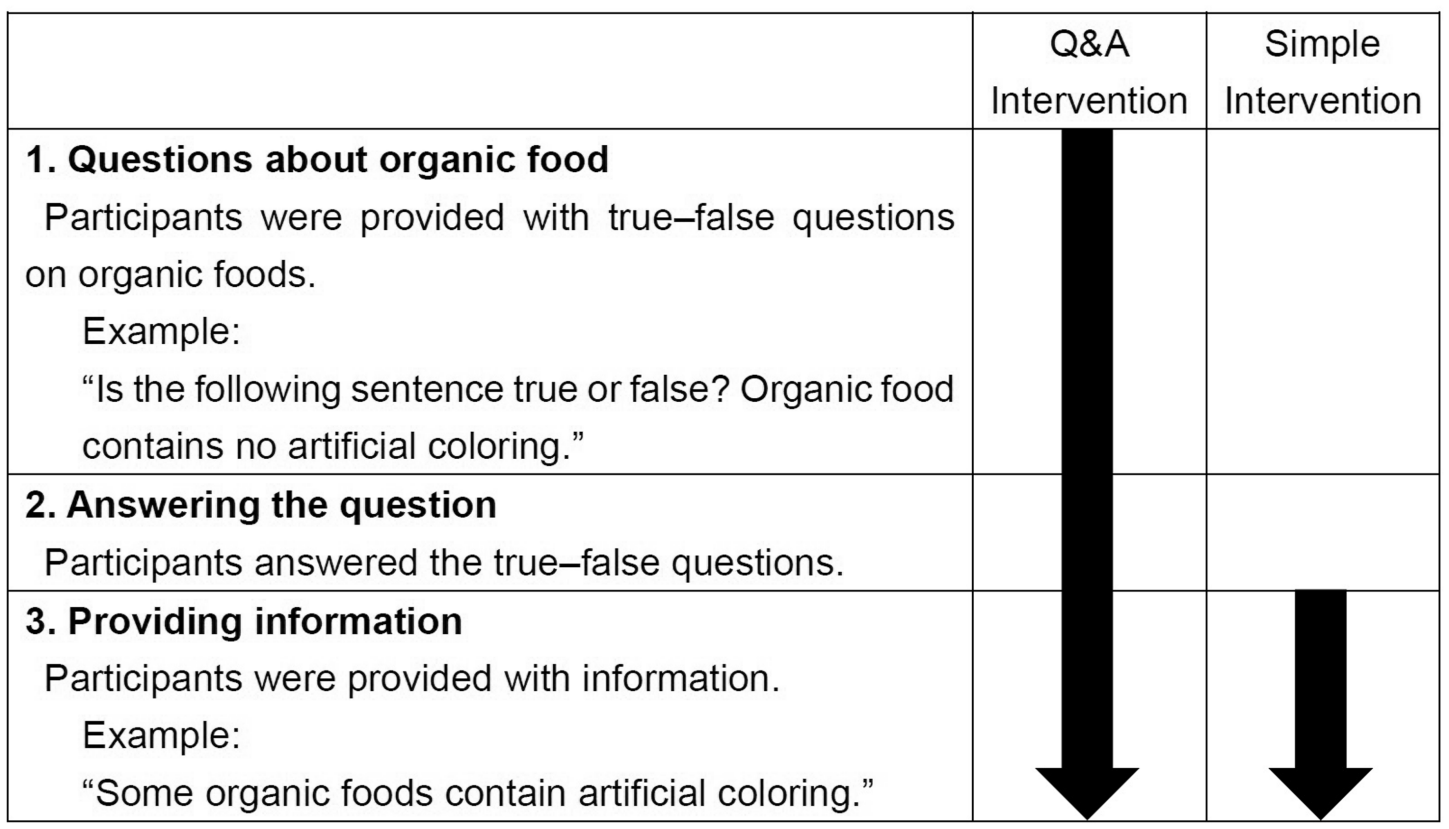
The process of Q&A intervention and simple intervention. Participants in Q&A intervention condition were provided with information after being asked to answer some questions and receiving feedback on whether their answers were correct. In this example, participants were informed: “Your answer was correct (incorrect). Some organic foods contain artificial coloring.”

We implemented two intervention methods (Figure 1): Q&A Intervention and Simple Intervention. We conducted Q&A Intervention based on Rozenblit and Keil (2002), which focused on self-assessed knowledge about the capitals of countries (the belief that one knows the names of the capital cities of various countries). First, their participants were asked to rate their self-assessed knowledge (i.e., how well they believed they knew the capitals of 48 countries). Thereafter, they were asked to state the name of each country’s capital city and were later provided with the correct answers. Through such Q&A Intervention, participants became aware whether they had the correct knowledge about the name of the capital city of each country. They were then asked to rate their self-assessed knowledge again. Their self-assessed knowledge after Q&A Intervention was lower than before the intervention. In our Q&A Intervention, as in their study, we (1) presented a question about organic foods, (2) asked the participants to answer it, and (3) informed them of the correct answer. Meanwhile, in Simple Intervention, we simply provided knowledge [i.e., we only conducted procedure (3)].

Based on Fernbach et al.’s (2013) study, we compared the participants’ self-assessed knowledge and attitude extremity before and after the intervention. We measured their attitudes through their willingness to pay (WTP) for organic foods.

### 2.1 Participants

Participants were selected through target sampling. Our study attempted to examine the intervention to moderate not only people’s extremely negative attitudes but also their extremely positive attitudes. If we conducted random sampling of Japanese, it would be difficult to recruit people with extremely positive attitudes and to examine whether our intervention could moderate their attitudes. This is because the organic share of total food retail sales in Japan is as low as 1.4% (Willer and Lernoud, 2019) and not a large proportion of Japanese individuals seems to have extremely positive attitudes toward organic foods.

Instead, we conducted target sampling to recruit participants with positive attitudes toward organic foods; only recruiting college-educated women. This is because, in Japan, college graduates or women are more likely to purchase organic foods than middle and high school graduates or men (Yasui, 2018). According to Yasui (2018), around 60% of college educated women answered “4: agree” or “3: slightly agree” to the question that they bought organic, pesticide-free, or additive-free foods. This percentage was higher than that of middle and high school graduated men (Yasui, 2018). To recruit those who had positive attitudes toward organic foods as well as those who had negative attitudes toward the foods, we recruited 716 college-educated women aged between 22 and 69 years through a research company, Rakuten Insight^1^ in 2020.

We excluded 28 people who did not answer all the questions, 6 who were judged not to have read the questions (more details in the Procedure section), and 2 people who had the same IP address. Since one participant answered they were a man, although we recruited from female monitors, we excluded this participant. We also excluded one respondent who made an unrealistic answer (whose WTP for strawberry jam was ¥500,000, which amounts to around US$ 5,000). We calculated the outliers through the Smirnov–Grubbs test and excluded 25 people. The total number of participants analyzed was 653, of which 327 were provided with knowledge after asking them some questions (Q&A Intervention), and 326 were simply provided with knowledge (Simple Intervention). The average age was 45.25 (*SD* = 10.86).

We examined whether the sample size was large enough using G*Power version 3.1.9.7 (Faul et al., 2007). We conducted a post-hoc power analysis, assuming *f* = 0.10 (small effect size), *α* = 0.05, *N* = 653, and the correlation among the repeated measures = 0.10 (low correlation). As a result, the calculated power of the test was 0.97, indicating an adequate sample size.

### 2.2 Experimental design

We conducted a two-factor mixed experimental design with measurement timing (within-participants variable: before or after knowledge provision) × intervention method (between-participants variable: Q&A Intervention or Simple Intervention).

The first variable was the timing of the measurement of self-assessed knowledge and the attitude of the participants (before or after the intervention; within-participants design). The second variable was the intervention method (Q&A Intervention or Simple Intervention; between-participants design). We hypothesized that Q&A Intervention would reduce their self-assessed knowledge and extremity of attitude, whereas Simple Intervention would not.

The experimental procedure was approved by the Ethics Review Committee for Experimental Research with Human Subjects at University of Tokyo. The study conformed to the protocol outlined in the latest version of the Declaration of Helsinki.

#### 2.2.1 Agreement

Participants were asked to provide written informed consent for participation in the study after being informed about the experiment’s purpose. This study was conducted online, and all participants indicated their consent to engage in the study.

#### 2.2.2 Instructional manipulation check

We checked whether participants had read the questionnaire carefully. We asked them to select “apple” and “orange” among the following choices: watermelon, kiwi, grape, banana, apple, melon, strawberry, mango, orange, and grapefruit. Those who did not accurately select these two fruits were not allowed to answer the following questions and were excluded from the analysis.

#### 2.2.3 WTP for organic foods (before intervention)

**Figure 2:**
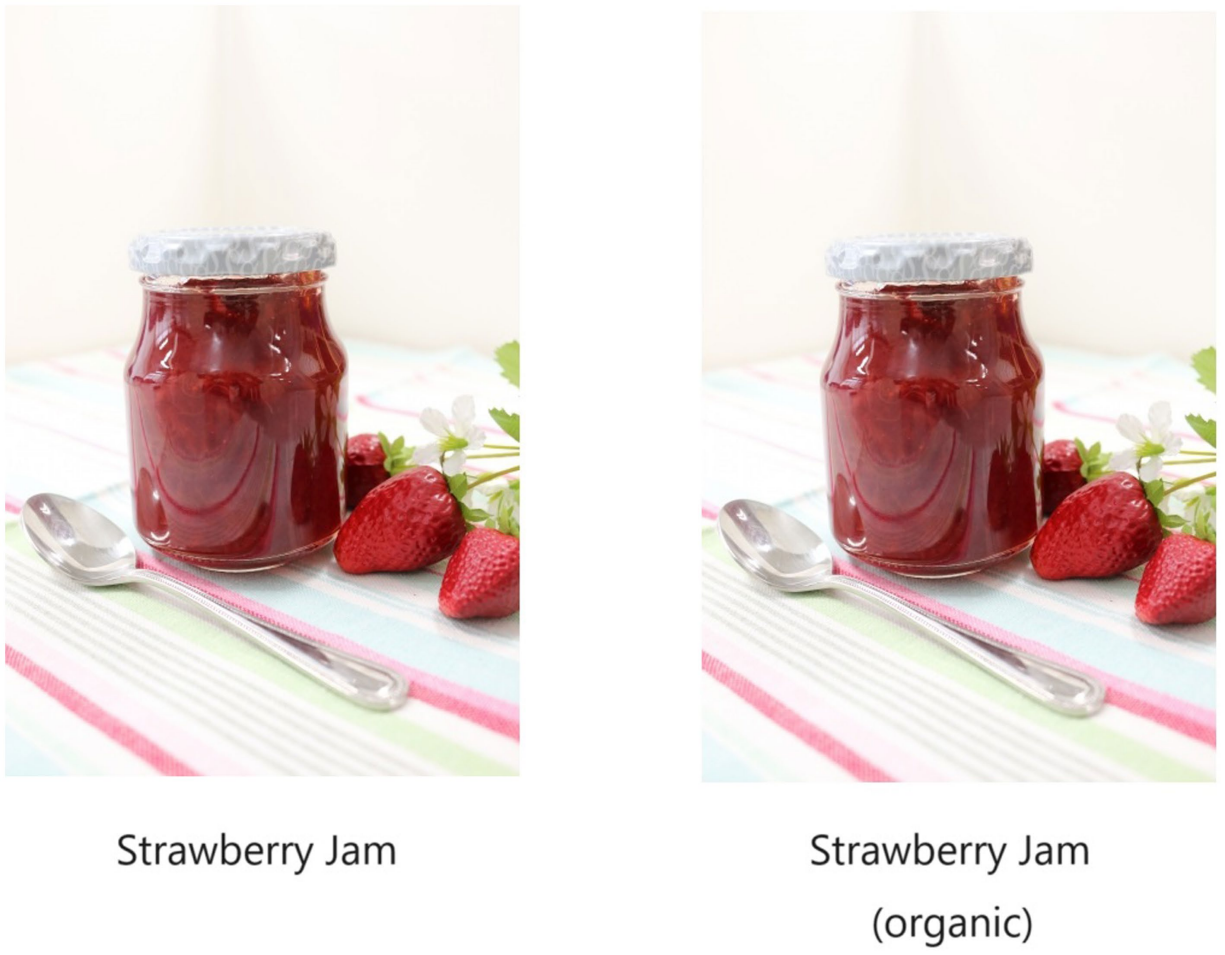
Example of food image with or without the text description of organic. Reproduced with permission from https://www.photo-ac.com/.

We asked participants to answer their WTP for organic foods. WTP has been utilized to measure people’s attitudes (e.g., Kahneman et al., 1990). We used this indicator to measure attitudes toward organic foods. The participants were presented with two images of food (Figure 2). These two images were identical except for the text description; one image had a description of “organic,” and the other image had no description. We asked the participants how much they were willing to pay for the food with the text description of organic when the food without the description cost a certain amount. In the example shown in Figure 2, participants were asked how much they were willing to pay for strawberry jam with the text description of organic when the jam without the organic description cost ¥200. The name of each food item and the baseline price (the price of foods without the descriptions) are shown in Table 1. The images of the foods other than strawberry jam are presented in Supplementary Figures S1–S4. Due to copyright issues, the presented image of tofu (Supplementary Figure S4) was not the same as those used in our experiment. These 5 foods were selected from 14 kinds of organic foods placed in some supermarkets in Tokyo. The prices of the food without the description were accurate representations of prices in supermarkets in Japan.

**Table 1:** The foods used in the study and their prices without the organic text description.

Food item	Price of the food item without the text description of organic (in Japanese yen)
Strawberry jam (160 g)	¥200
Nuts (150 g)	¥400
Carrots (three sticks)	¥200
Miso (500 g)	¥300
Tofu (one pack)	¥150

#### 2.2.4 Self-assessed knowledge of organic foods (before intervention)

The participants were asked to answer how familiar they were with organic foods. They responded using a visual analog scale ranging from 0 (do not know at all) to 100 (know a lot about it). We added the following explanations to convey the level of understanding which each value represents: “Those with a level 100 understanding are familiar with the details of organic foods,” “Those with a level 50 understanding have some knowledge of organic foods,” and “Those with 0 understanding have never heard of the term organic foods.”

#### 2.2.5 Intervention

Participants in Q&A Intervention condition were asked to answer five true–false questions (see Table 2) after they agreed to answer them without consulting the internet or books. We created the questions (1) which only included everyday languages so that participants could understand each word in the questions, but (2) which not a few people were estimated to misunderstand. We created questions regarding the use of pesticides (Question 1), nutrition (Question 2), food labels (Question 3), and food additives (Questions 4 and 5) in organic foods. Research demonstrated that one of the main motives for buying organic foods was their perceived benefit for human health (Magnusson et al., 2003; Chen, 2009), and indeed, around 80% of Japanese consumers thought that organic foods which they bought were good for health (Ministry of Agriculture, Forestry and Fisheries, 2018). Therefore, Japanese consumers were estimated to misunderstand that organic foods had more nutrition and no food additives. The answers to questions 1, 3, 4, and 5 were created based on Japan Agricultural Standards for Organic Agricultural Products (Ministry of Agriculture, Forestry and Fisheries, 2022).^2^ The answer to question 2 was created based on Dangour et al. (2009).

**Table 2:** Questions and answers.

	**Question**	**Answer**	**Percentages of correct answers**
1	The use of some kinds of pesticides is allowed when growing organic vegetables.	True	33.94%
2	Foods labeled as organic have more nutrition than those that are not.	False	80.12%
3	The conditions for labeling “オーガニック野菜 (organic vegetable)” are different from the conditions for labeling “有機野菜 (vegetables with organic compounds).”	False	29.66%
4	Some organic foods contain preservatives.	True	65.14%
5	Organic foods contain no artificial coloring.	False	63.91%

We set the answers to Questions 1, 3, 4, and 5 based on legally determined standards in Japan.

**Figure 3:**
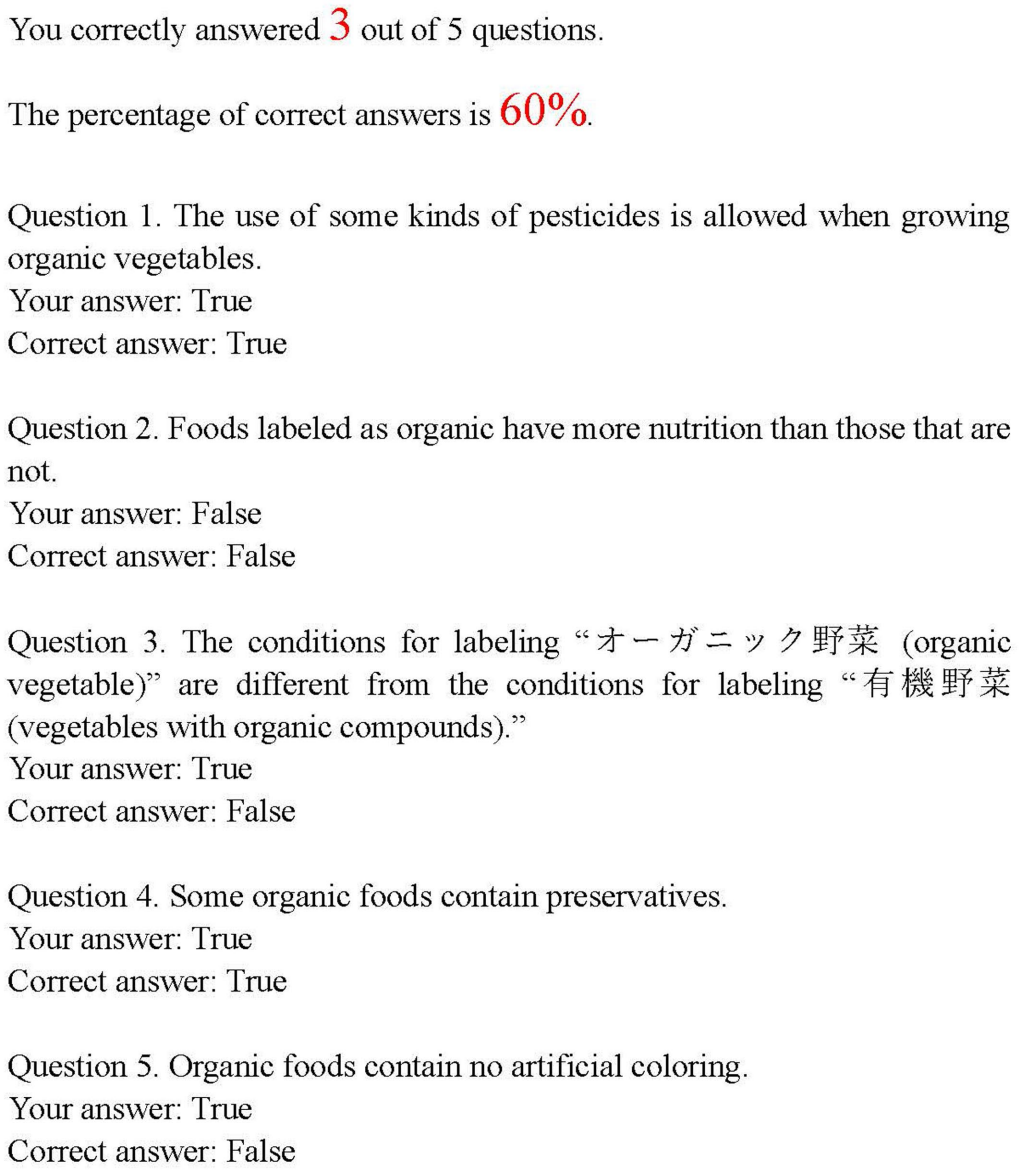
Feedback regarding correct answers.

Every time the participants answered a question, they were informed of the correct answer and whether their answer was correct. After they answered all the questions, they were shown their answers, the correct answers, and the percentage of correct answers (Figure 3). Participants in Simple Intervention condition were presented with the same five knowledge statements on organic food, with the same correct answers as those in Q&A Intervention condition (e.g., the use of some kinds of pesticides is allowed when growing organic vegetables).

#### 2.2.6 Self-assessed knowledge of organic foods (after intervention)

After the intervention, we measured self-assessed knowledge of organic foods again. We asked participants to reflect on their self-assessed knowledge before the intervention by asking the following question: “Looking back now, how familiar were you with organic foods before this experiment?” As in the first measurement, participants responded using a visual analog scale ranging from 0 (do not know at all) to 100 (know a lot about it).

#### 2.2.7 WTP for organic foods (after intervention)

Finally, we asked a question to measure the participants’ attitudes toward organic foods again. As in the first measurement, we asked them how much they were willing to pay for food with the text description of “organic” when food with no description cost a certain amount.

## 3 Results

As shown in Table 2, the percentage of correct answers was lower than 40% in Questions 1 and 3. The rate was around 65% for Questions 4 and 5. These results implied that not a few participants had incorrect knowledge of the statements in Questions 1, 3, 4, and 5, although, contrary to our estimation, most participants had correct knowledge of the statement in Question 2.

### 3.1 Did the intervention reduce participants’ self-assessed knowledge?

We conducted a two-way analysis of variance (ANOVA) to test whether our interventions reduced self-assessed knowledge. The independent variables were the intervention method (Q&A Intervention or Simple Intervention) as a between-participants factor and measurement timing (before or after intervention) as a within-participants factor. Self-assessed knowledge was the dependent variable.

**Figure 4:**
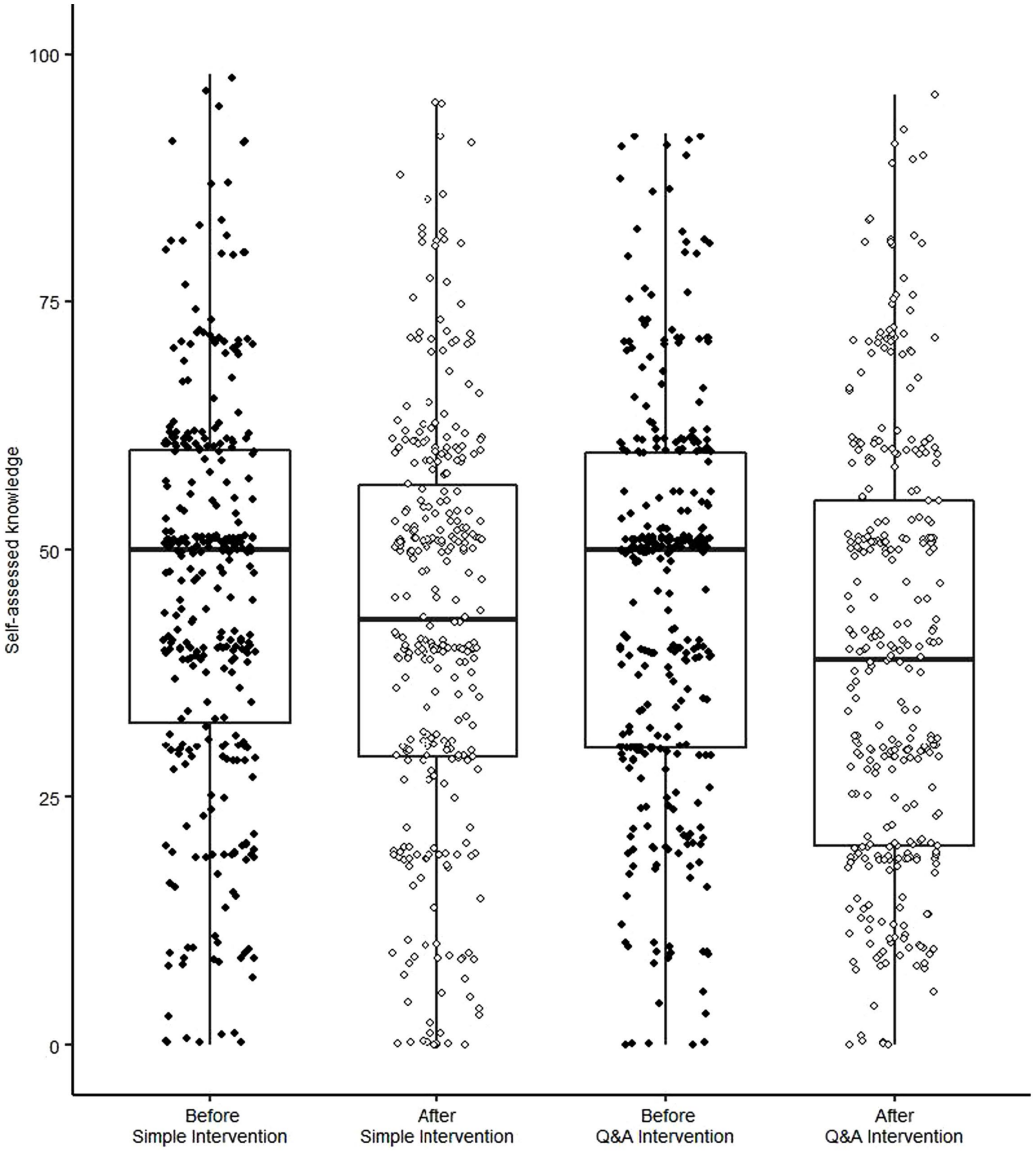
Box plots of self-assessed knowledge before and after intervention.

As shown in Figure 4, the main effect of measurement timing was statistically significant [*F* (1, 651) = 36.46, *p* < 0.001, η^2^_p_ = 0.05], indicating that participants’ self-assessed knowledge after the intervention was lower than that before it. This effect size was larger than 0.04, the “recommended minimum effect size representing a ‘practically’ significant effect for social science data” (Ferguson, 2009). The interaction effect between the intervention and measurement timing was not significant [*F*(1, 651) = 1.91, *p* = 0.17, η^2^_p_ = 0.00]. These results implied that Simple Intervention, as well as Q&A Intervention, on average, reduced participants’ self-assessed knowledge. Indeed, regardless of the intervention style, self-assessed knowledge after intervention (*M* = 42.06, *SD* = 21.63 for Q&A Intervention, *M* = 39.26, *SD* = 22.16 for Simple Intervention) was lower than that before the intervention (*M* = 45.46, *SD* = 20.12 for Q&A Intervention; *M* = 44.68, *SD* = 20.21 for Simple Intervention). The main effect of the intervention method [*F* (1, 651) = 1.47, *p* = 0.23, η^2^_p_ = 0.00] was not significant.

### 3.2 Did the intervention moderate the extremity of participants’ attitudes toward organic foods?

Next, we focused on participants’ attitudes toward organic foods. As shown in Table 3, their WTP for organic foods was higher than the food item price without the organic text description. This implied that participants generally had positive attitudes toward organic foods.

**Table 3:** Participants’ WTP before and after intervention.

**Food item**	**Price of the food item without the text description of “organic”**	**WTP** **before Q&A Intervention**	**WTP** **after Q&A Intervention**	**WTP** **before Simple Intervention**	**WTP** **after Simple Intervention**
**Strawberry jam (160 g)**	¥200	301.33 (88.62)	270.29 (79.62)	304.83 (96.99)	265.89 (77.21)
**Nuts (150 g)**	¥400	490.97 (109.27)	464.33 (107.42)	493.23 (109.35)	458.56 (87.88)
**Carrots (three sticks)**	¥200	261.46 (62.71)	247.64 (61.02)	259.54 (58.45)	241.18 (50.04)
**Miso (500 g)**	¥300	415.58 (116.61)	383.16 (99.50)	405.73 (109.58)	371.34 (92.82)
**Tofu (one pack)**	¥150	199.76 (53.74)	191.81 (56.45)	197.40 (50.80)	185.84 (48.89)

Values in parentheses indicate standard deviation. ¥, Japanese yen.

We conducted a two-way ANOVA to test whether our intervention moderated participants’ attitude extremity. The independent variables were the intervention method and measurement timing. The dependent variable was the extremity of their attitude toward each organic food item.

The absolute difference between their WTP for a food item with the organic description and the food item price without the description was operationally defined as the extremity of their attitude. This definition was adapted from Fernbach et al. (2013), who measured participants’ political attitudes using a seven-point scale and defined the absolute difference between their attitude and the midpoint (4 points) as the extremity of the attitude. Since our study measured the extremity of individuals’ attitudes by measuring WTP, which has no midpoint, we set the food item price without the organic description as the baseline. WTP would be higher than the baseline price among those with positive attitudes and vice versa. In addition, this difference would be larger among those with strongly positive (or negative) attitudes. For example, imagine a situation where the price of a non-organic food item was ¥500. WTP among participants with extremely positive attitudes would be much larger than the baseline price (e.g., ¥800); in comparison, WTP among participants with moderately positive attitudes would be moderately larger than the baseline price (e.g., ¥600). Therefore, the difference between WTP and the baseline would be larger among those with extremely positive attitudes (¥300) than those with moderately positive attitudes (¥100). This is also the case among those with negative attitudes. Therefore, we calculated the absolute difference between WTP and the baseline price of non-organic food items and regarded this as their attitude extremity.

**Figure 5:**
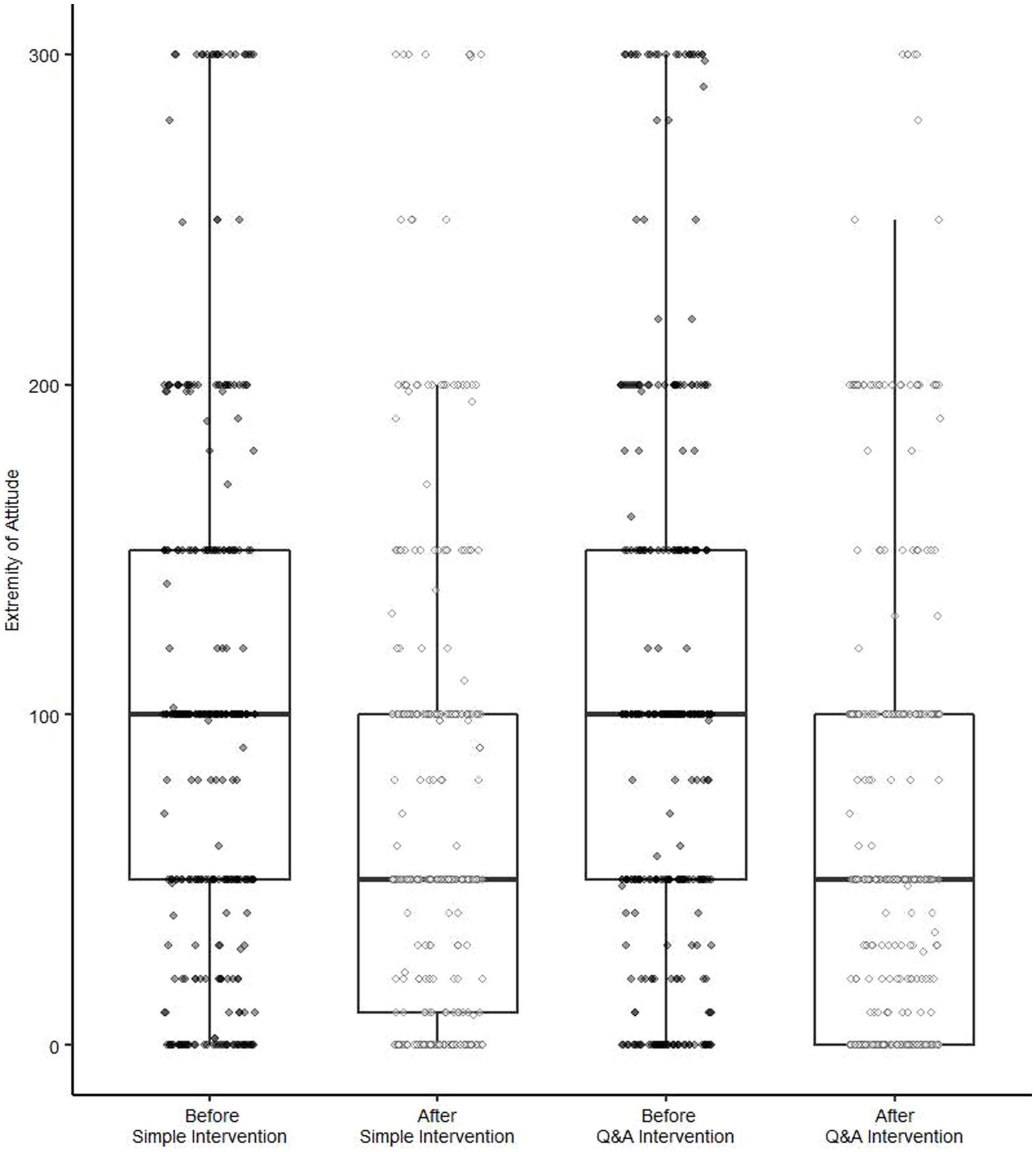
Box plots of the extremity of attitude for strawberry jam before and after intervention. The extremity of attitude was calculated as the absolute difference between the participants’ WTP for the strawberry jam with the text description of organic and the price of the jam without the description (¥200).

As shown in Figure 5 and Table 4, the main effect of measurement timing was statistically significant, which meant that the participants’ attitudes after the intervention were generally more moderate than prior to it [*F*(1, 651) = 184.54, *p* < 0.001, η^2^_p_ = 0.22 for strawberry jam; *F*(1, 651) = 110.00, *p* < 0.001, η^2^*_p_* = 0.14 for nuts; *F*(1, 651) = 95.58, *p* < 0.001, η^2^*_p_* = 0.13 for carrots; *F*(1, 651) = 141.75, *p* < 0.001, η^2^_p_ = 0.18 for miso; and *F*(1, 651) = 39.26, *p* < 0.001, η^2^_p_ = 0.06 for tofu].

**Table 4:** The extremity of participants’ attitude before and after intervention.

**Food item**	**Price of the food item without the text description of organic**	**Extremity of their attitude before Q&A intervention**	**Extremity of their attitude after Q&A intervention**	**Extremity of their attitude before simple intervention**	**Extremity of their attitude after simple intervention**
**Strawberry jam (160 g)**	¥200	103.63 (85.91)	76.01 (74.15)	108.98 (92.28)	70.55 (72.96)
**Nuts (150 g)**	¥400	106.61 (94.03)	86.88 (90.10)	106.00 (96.98)	70.93 (78.21)
**Carrots (three sticks)**	¥200	66.60 (57.20)	55.18 (54.27)	64.12 (53.36)	46.21 (45.42)
**Miso (500 g)**	¥300	121.21 (110.73)	91.02 (92.35)	111.68 (103.49)	78.40 (86.93)
**Tofu (one pack)**	¥150	54.32 (49.11)	47.68 (51.58)	51.34 (46.81)	39.96 (45.58)

The extremity of their attitude is the difference between their WTP for the food item with the text description of organic and the price of the food without the description. Values in parentheses indicate standard deviation. ¥, Japanese yen.

The main effect of the intervention method was not significant [*F*(1, 651) = 0.00, *p* = 0.99, η^2^_p_ = 0.00 for strawberry jam; *F*(1, 651) = 1.60, *p* = 0.21, η^2^_p_ = 0.00 for nuts; *F*(1, 651) = 2.21, *p* = 0.14, η^2^_p_ = 0.00 for carrots; *F*(1, 651) = 2.33, *p* = 0.13, η^2^_p_ = 0.00 for miso; and *F*(1, 651) = 2.33, *p* = 0.13, η^2^_p_ = 0.00 for tofu].

The interaction effect between measurement timing and intervention method was significant in some food items [*F*(1, 651) = 4.94, *p* < 0.03, η^2^_p_ = 0.01 for strawberry jam; *F*(1, 651) = 8.63, *p* < 0.01, η^2^_p_ = 0.01 for nuts; and *F*(1, 651) = 4.67, *p* < 0.04, η^2^_p_ = 0.01 for carrots] although the effect size was as weak as 0.01. The simple main effect of measurement timing on moderating attitude extremity was larger among participants in Simple Intervention condition [*F*(1, 325) = 117.81, *p* < 0.001, η^2^_p_ = 0.27 for strawberry jam; *F*(1, 325) = 92.35, *p* < 0.001, η^2^_p_ = 0.22 for nuts; *F*(1, 325) = 61.06, *p* < 0.001, η^2^_p_ = 0.16 for carrots] than among participants in Q&A Intervention condition [*F*(1, 326) = 68.68, *p* < 0.001, η^2^_p_ = 0.17 for strawberry jam; *F*(1, 326) = 27.84, *p* < 0.001, η^2^_p_ = 0.08 for nuts; *F*(1, 326) = 34.77, *p* < 0.001, η^2^*_p_* = 0.10 for carrots]. However, the interaction effect was not significant in other foods [*F*(1, 651) = 0.34, *p* = 0.56, η^2^_p_ = 0.00 for miso; and *F*(1, 651) = 2.71, *p* = 0.10, η^2^_p_ = 0.00 for tofu].

Since age was positively associated with one’s attitudes toward organic foods (Yasui, 2018), we conducted an additional analysis that controlled for the effect of age. The results were the same as those reported above. The method and results of the additional analysis are provided in Supplementary material S2.

## 4 Discussion

### 4.1 Results summary and theoretical implications

We conducted interventions to reduce people’s self-assessed knowledge about organic foods and moderate the extremity of their attitude toward these foods. We conducted two interventions: one that provided knowledge after asking them some questions (Q&A Intervention) and the other that simply provided knowledge without asking them any questions (Simple Intervention). Inconsistent with our hypotheses, both intervention methods, on average, decreased participants’ self-assessed knowledge and moderated their attitudes.

Why was there no difference in the effect of the intervention method on moderating people’s attitude extremity between Simple Intervention and Q&A Intervention? We can speculate on at least three reasons. The first reason is that both intervention methods reduced people’s self-assessed knowledge. Indeed, regardless of the intervention method, self-assessed knowledge after the intervention was lower than that before the intervention. The second reason is that both intervention methods increased people’s actual knowledge. Participants might have accepted the provided knowledge even without being asked to answer any questions, and thus, their attitude extremity was weakened. However, we cannot examine this possibility because we cannot measure participants’ actual knowledge before Simple Intervention. The third reason is that Q&A Intervention puts a cognitive load on the participants. People make more affective decisions, rather than rational decisions, when their cognitive resources are limited (Shiv and Fedorikhin, 1999). Therefore, if participants in Q&A Intervention condition experienced more cognitive strain than those in Simple Intervention condition, they would make more extreme judgments. Those with more positive attitudes toward organic foods would answer higher WTP and vice versa. Indeed, Q&A Intervention less effectively moderated people’s attitude extremity toward some foods than Simple Intervention (the interaction effect). Although we tried to moderate their attitude extremity through Q&A Intervention, this effect might be canceled out partially because Q&A Intervention put more cognitive strain and made their attitude more extreme.

Our intervention was closely related to that in the testing effect (Roediger III and Karpicke, 2006). The testing effect refers to “the phenomenon of improved performance from taking a test” (Roediger and Karpicke, 2006, p. 181). People’s memory is enhanced when they are forced to take tests rather than when they simply read some information (Butler, 2010). Therefore, Q&A Intervention can weaken people’s extreme attitudes not only by further reducing their self-assessed knowledge but also by letting them memorize the provided information better than Simple Intervention. Indeed, Q&A Intervention, on average, succeeded in moderating people’s extreme attitudes, but Simple Intervention also moderated their attitudes as much as Q&A Intervention did. Future studies should examine whether asking some questions before knowledge provision is essential for weakening people’s extreme attitudes toward other topics, such as attitudes toward genetically modified foods or vaccines.

### 4.2 Implications for consumer behavior

In this study, we focused on organic foods. Some studies have demonstrated their positive attitudes toward organic foods among consumers in the United States or Italy. Lee et al. (2013) surveyed consumers in New York and demonstrated that they tend to believe organic-labeled foods contain more nutrition. Richetin et al. (2022) surveyed individuals in Italy and demonstrated that they perceived organic-labeled cookies as healthier even when the foods were less healthy (i.e., having more fat and sugar) than non-labeled foods. Considering that research is inconclusive on the positive effects of organic foods on human health, consumers who believe organic foods to be healthy may have extremely positive attitudes toward such foods.

By contrast, other studies have demonstrated negative attitudes toward organic foods among consumers in the United States or Belgium. The studies demonstrated that organic foods were perceived as less tasty among consumers in the United States (Schuldt and Hannahan, 2013) and in Belgium (Rousseau, 2015). In addition, Aschemann-Witzel and Zielke (2017)’s review paper claimed that consumers’ perceived price of organic foods was outdated and erroneously higher than actual. Those consumers who perceive organic foods as less tasty or excessively expensive might have extremely negative attitudes toward organic foods, although it needs to be noted that other studies demonstrated organic foods, especially healthy organic foods (e.g., organic apples or organic fresh orange juice), were perceived as tastier among consumers in the United States and Netherlands (Nadricka et al., 2020).

In this study, we tried to moderate people’s extremely positive and negative attitudes toward organic foods. We conducted two interventions: (1) simply providing knowledge on organic foods (Simple Intervention) and (2) providing knowledge after asking them some questions (Q&A Intervention). Both interventions were generally successful in moderating people’s extreme attitudes toward organic foods.

Our results have significant implications for consumer behavior. The first issue is *food faddism*. Food faddism is a phenomenon wherein people falsely believe in the effects of specific foods on health and disease (Jarvis, 1983). If people consume specific foods to prevent or cure diseases while rejecting scientific treatment, this may result in early death. For instance, some people have consumed natural foods to cure their diseases despite the lack of scientific evidence to support these beliefs (McBean and Speckmann, 1974). Therefore, it is important to reduce people’s extremely positive attitudes toward certain foods to prevent food faddism. Based on our results, there were at least two interventions to reduce people’s extremely positive attitudes and food faddism; (1) simply providing knowledge (e.g., providing knowledge that natural foods were not scientifically proven to cure diseases) and (2) providing knowledge after asking them a few questions (e.g., providing the abovementioned knowledge after asking them whether natural foods were scientifically proven to cure diseases).

Second, we can apply our findings to moderate people’s extremely negative attitudes toward other foods, for example, genetically modified food. Genetically modified food could contribute toward crop improvements such as increasing nutritional content or disease resistance (Sharma et al., 2017), and there is a scientific consensus that genetically modified foods are safe to eat (American Association for the Advancement of Science, 2012). However, more than half of the people surveyed by Pew Research Center (2015) perceived genetically modified foods to be unsafe for eating. Therefore, to utilize the potential of genetically modified foods, such as the potential to address food shortage problems through crop improvements, it is essential to reduce people’s extremely negative attitudes toward these foods. Based on our results, we believe, at least two interventions can be suggested to moderate their extremely negative attitudes toward genetically modified foods and to increase their consumption of the foods; (1) simply providing knowledge (e.g., knowledge that there is a scientific consensus that genetically modified foods are safe to eat) or (2) providing knowledge after asking them some questions (e.g., providing the abovementioned knowledge after asking them whether they believe genetically modified foods are safe to eat).

Third, our results demonstrated that simply providing knowledge also, on average, reduced participants’ self-assessed knowledge and moderated their attitude extremity. Information that contradicts people’s political preferences is less likely to be accepted by people (Sunstein et al., 2016), but organic foods are not related to their political preferences (Larson, 2018). This is why participants might have accepted the provided information. Therefore, providing factual knowledge will also be an effective intervention to change people’s attitudes toward some foods, such as fruits or vegetables, which have no political or ideological implications. For example, future campaigns could put more effort into increasing people’s factual knowledge regarding vegetables and fruits, such as the exact recommended amount of consumption. In a study involving 5 a day campaign (Herbert et al., 2010), although people were aware that they should eat at least five daily servings of fruits and vegetables through 5 a day campaign, they did not know what the five servings entailed. According to our results, simply providing knowledge (e.g., knowledge that “5 a day campaign” promotes individuals to consume five daily servings of fruits and vegetables, and this amount is equivalent to 400 g) can increase their consumption of fruits or vegetables.

### 4.3 Limitations

This study has some limitations. First, the validity of the operational definition of the extremity of attitude needs to be examined further. We defined the difference between the price of food without an organic description and participants’ WTP for food items with the text description of organic as the extremity of their attitude. This definition is based on Fernbach et al. (2013). Future studies need to examine the validity of this operational definition.

Second, participants’ WTP may not reflect their true preferences. Participants in this study did not need to buy organic foods; in a hypothetical situation, participants tended to report higher-than-actual WTP values (Lusk and Schroeder, 2004). This bias could be reduced through actual payment settings (Miller et al., 2011), wherein participants need to buy items when the price is cheaper than their stated WTP (Becker et al., 1964). However, conducting an actual-payment-setting experiment is expensive, as it requires sending the items (organic foods) to some of the participants. Further, according to Miller et al. (2011), there is no difference between participants’ actual WTP and the WTP stated in hypothetical settings. Therefore, although WTP measured in hypothetical settings might be biased, we believe that this bias does not hurt the validity of our findings.

Third, our criterion for target sampling might not have been adequate for recruiting people with positive attitudes. We recruited college-educated women to include both people with positive attitudes toward organic foods and people with negative attitudes. However, education and gender are not the only factors that affect people’s tendency to buy organic foods. For example, people with higher equivalent household income (value of household income divided by the square root of the number of individuals in the household) are more likely to buy organic foods (Yasui, 2018). Future studies should adopt stricter criteria for recruiting people with positive attitudes toward organic foods.

Fourth, we did not manipulate the food label. We used the same pictures for organic and non-organic food and compared the food with or without the text description “organic.” This is because food labels are rarely put directly on some foods (e.g., carrots). Future studies should manipulate the food labels, manipulating pictures on food labels as well as text descriptions because consumers react both to text descriptions and pictures (Tang et al., 2004). This manipulation can also increase the reality and the practical implications.

Finally, although Simple Intervention generally succeeded in moderating people’s attitudes toward foods in our study, this intervention will not succeed when the foods are related to some political preferences. People often do not believe in information that differs from their ideology (Sunstein et al., 2016). Therefore, US Republicans, who are less supportive of fair trade products than Democrats (Park, 2018), might not accept knowledge of fair trade products as it is, especially knowledge of the goodness of fair trade products. Future studies should examine whether Q&A Intervention is more effective than Simple Intervention in moderating people’s attitudes toward foods which are related to political preferences.

## 5 Conclusion

In this study, we examined the intervention to reduce self-assessed knowledge of organic foods and to moderate people’s extremely positive or negative attitudes toward organic foods. We implemented two kinds of interventions. The first was Simple Intervention; simply providing information on organic foods. The second was Q&A Intervention; providing information after asking them some questions. We found that Simple Intervention, as well as Q&A Intervention, reduced participants’ self-assessed knowledge and extremity of their attitudes toward these foods. This result implies that simply providing knowledge is effective in lowering self-assessed knowledge and modifying attitudes toward food.

We also discussed the limitation of Simple Intervention. Participants in this study accepted the knowledge and lowered their self-assessed knowledge, even without being asked to answer some questions before knowledge provision. This might be because the provided knowledge (i.e., knowledge on the definition of organic foods) is a non-political or ideological topic. Participants may not accept knowledge having political implications, such as the goodness of fair trade products, because people frequently do not trust information that contradicts their ideology. In such cases, Q&A Intervention, which involves asking questions before providing knowledge, would be more effective than Simple Intervention.

## Data availability statement

The original contributions presented in the study are included in the article/Supplementary material, Data and R code are publicly available via the Open Science Framework and can be accessed at https://osf.io/6bm8y/?view_only=6086ceaa19dc43eabd0f5150f2b6e994. Further inquiries can be directed to the corresponding authors.

## Ethics statement

The studies involving humans were approved by the Ethics Review Committee for Experimental Research with Human Subjects at the University of Tokyo. The studies were conducted in accordance with the local legislation and institutional requirements. The participants provided their written informed consent to participate in this study.

## Author contributions

SI: Conceptualization, Data curation, Funding acquisition, Formal analysis, Investigation, Methodology, Visualization, Writing – original draft. HH: Conceptualization, Investigation, Methodology, Writing – review & editing. YO: Conceptualization, Investigation, Methodology, Writing – review & editing. KU: Conceptualization, Funding acquisition, Investigation, Methodology, Project administration, Supervision, Writing – review & editing.

## Footnotes

^1^ https://insight.rakuten.co.jp/en/

^2^Our questions were created based on Japan Agricultural Standards for Organic Agricultural Products in 2017. We cited the standard in 2022 because the standard in 2017 was not found online. The standard in 2017, which is written in Japanese, is presented at Supplementary material S3.
