# Supplementary material for: Effect of asking questions and providing knowledge on attitudes toward organic foods among Japanese consumers
Source: Front Psychol. 2023 Dec 27;14:1274446. doi: 10.3389/fpsyg.2023.1274446 (PMC10779989; doi:10.3389/fpsyg.2023.1274446)
Supplement: Supplementary file 1 [file Data_Sheet_1.DOCX]

Supplementary Material

# [Supplementary Material 1 is submitted separately as figures]

# 1 Supplementary Material 2

We examined whether knowledge provision made participants’ attitudes toward organic foods more moderate. We conducted additional analysis, which controlled for the effect of age. Age was entered as an independent variable because older people might have extremely positive attitudes toward organic foods (Yasui 2018). After mean-centering all the variables in the model, we conducted a generalized linear mixed model with intervention method (Q&A Intervention = 1, Simple Intervention = 0), knowledge provision (after intervention = 1, before intervention = 0), the interaction of these two variables, and age as independent variables. The dependent variable was the extremity of participants’ attitudes; based on the findings of Fernbach et al.’s (2013) study, the difference between individuals’ willingness to pay for a food item with an organic label and for a food item without an organic label was operationally defined as the extremity of their attitude.

The main effect of knowledge provision was statistically significant in this additional analysis. This suggests that the participants’ attitudes after intervention was more moderate than before it (β = -.20, *p* < .001 for strawberry jam; β = -.15, *p* < .001 for nuts; β = -.14, *p* < .001 for carrots; β = -.16, *p* < .001 for miso; and β = -.09, *p* < .001 for tofu). However, the main effect of the intervention method was not significant (β = .00, *p* = .99 for strawberry jam; β =.05, *p* = .21 for nuts; β = .05, *p* = .14 for carrots; β = .06, *p* = .13 for miso; and β = .05, *p* = .13 for tofu). Age was not related to the extremity of their attitudes (β = .01, p = .71 for strawberry jam; β = .01, p = .85 for nuts; β = .02, p = .59 for carrots; β = .02, p = .56 for miso; and β = .02, p = .66 for tofu). The interaction effect between knowledge provision and the intervention method was significant for some food items (β =.03, *p* < .03 for strawberry jam; β = .04, *p* < .01 for nuts; and β = .03, *p* < .04 for carrots), but not significant for others (β = .01, *p* = .56 for miso; and β = .02, *p* = .10 for tofu). Considering that the effect size of β was .04 at most, we did not interpret this effect.

**2 Supplementary Material 3**

We also examined whether knowledge provision attenuated the relationship between self-assessed knowledge and the extremity of attitudes toward organic foods. In the additional analysis, we controlled for the effect of age. After mean-centering all the variables in the model, we regressed willingness to pay after the intervention on a dummy variable of intervention method (Q&A Intervention = 1, Simple Intervention = 0), self-assessed knowledge before the intervention, the interaction of these two variables, and age. The dependent variable was the extremity of attitude, with the same aforementioned operational definition.

The main effect of self-assessed knowledge was robustly statistically significant in all the analyses, which meant that people with higher self-assessed knowledge had a more extreme attitude (β = .24, *p* < .001 for strawberry jam; β = .22, *p* < .001 for nuts; β = .23, *p* < .001 for carrots; β = .25, *p* < .001 for miso; and β = .19, *p* < .001 for tofu). The main effect of the intervention method was statistically significant for some food items (β = .09, *p* < .02 for nuts and β = .08, *p* < .03 for carrots), which indicated that the attitudes of the participants in the Simple Intervention condition were less extreme than those of the participants in the Q&A Intervention condition. Contrarily, this effect was not significant for other food items (β = .03, *p* = .40 for strawberry jam; β = .07, *p* = .09 for miso; and β = .08, *p* = .05 for tofu); thus, this effect was not robust. The interaction effect was not found for any kinds of food items (β = .02, *p* = .57 for strawberry jam; β = .02, *p* = .57 for nuts; β = .01, *p* = .77 for carrots; β = .01, *p* = .73 for miso; and β = .01, *p* = .74 for tofu). The main effect of age was also not found (β = .00, *p* = .94 for strawberry jam; β = -.02, *p* = .64 for nuts; β = .03, *p* = .49 for carrots; β = .01, *p* = .71 for miso; and β = .00, *p* = .92 for tofu).
