# Supplementary figures and images for: Effect of asking questions and providing knowledge on attitudes toward organic foods among Japanese consumers
Source: Front Psychol. 2023 Dec 27;14:1274446. doi: 10.3389/fpsyg.2023.1274446 (PMC10779989; doi:10.3389/fpsyg.2023.1274446)

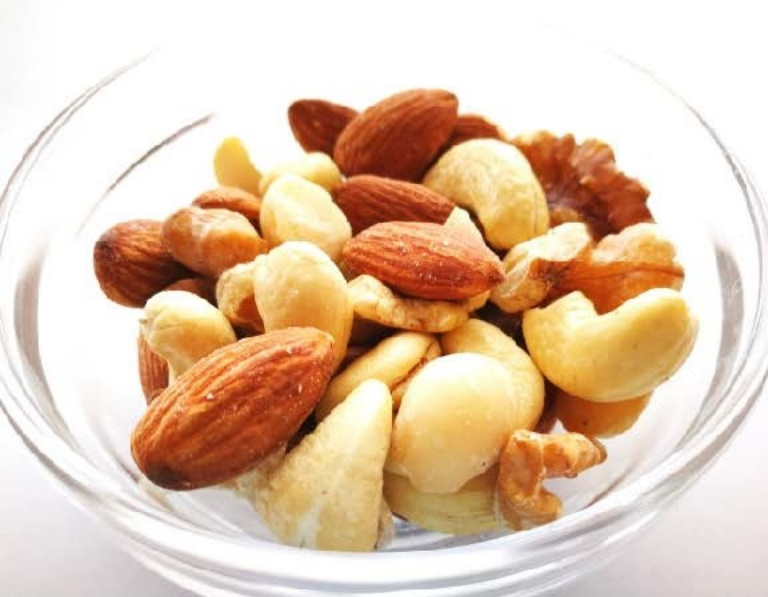

Nuts

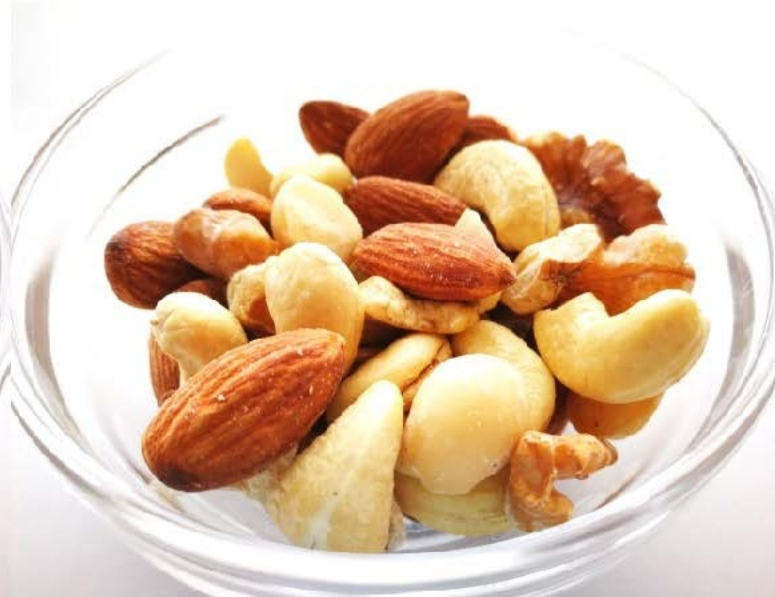

Nuts  
(organic)

Supplement: Supplementary file 2 [file Image_1.pdf]

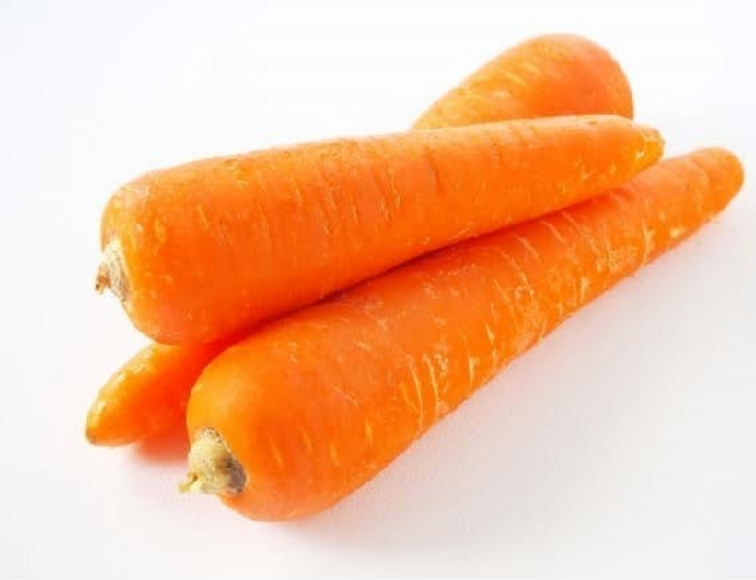

Carrots

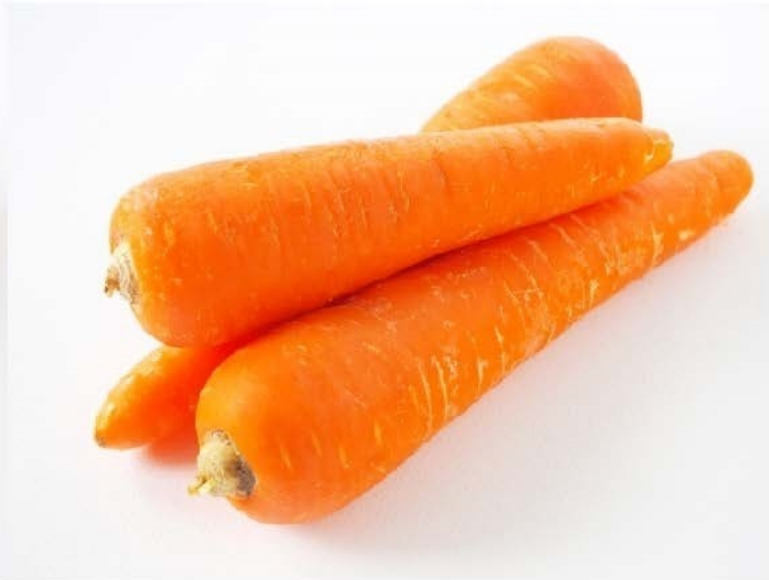

Carrots  
(organic)

Supplement: Supplementary file 3 [file Image_2.pdf]

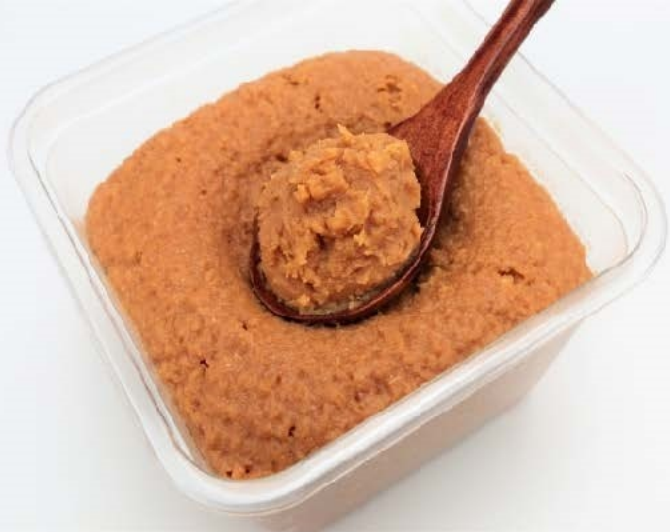

Miso

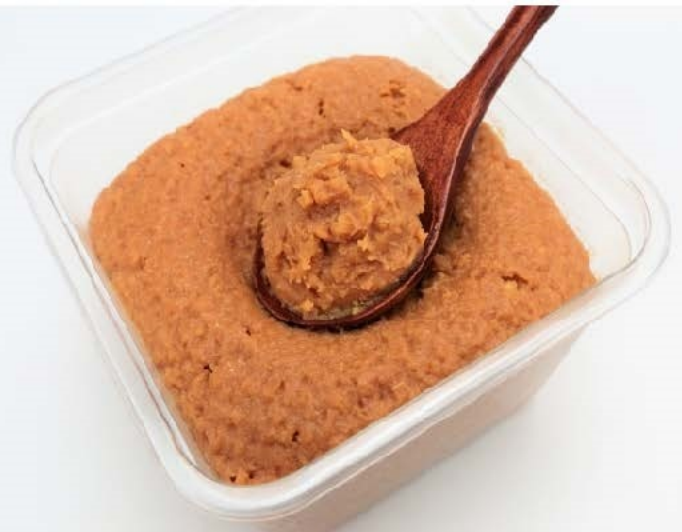

Miso  
(organic)

Supplement: Supplementary file 4 [file Image_3.pdf]

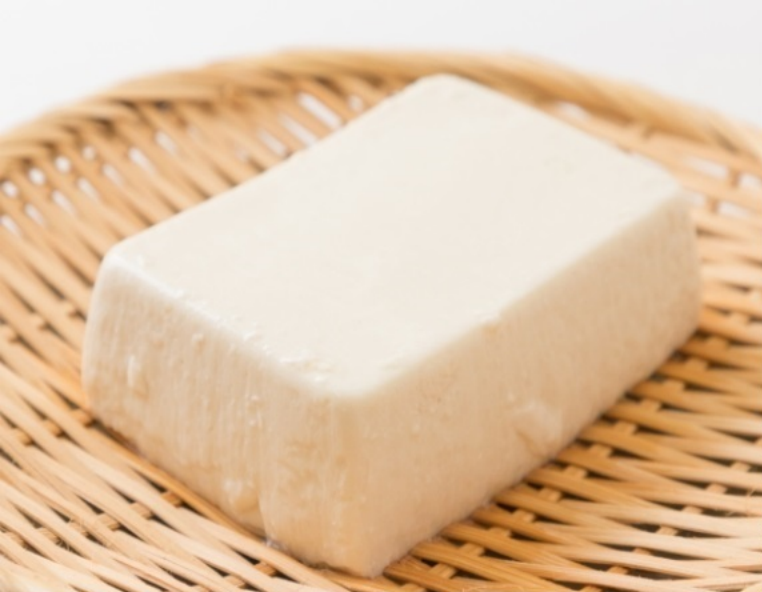

Tofu

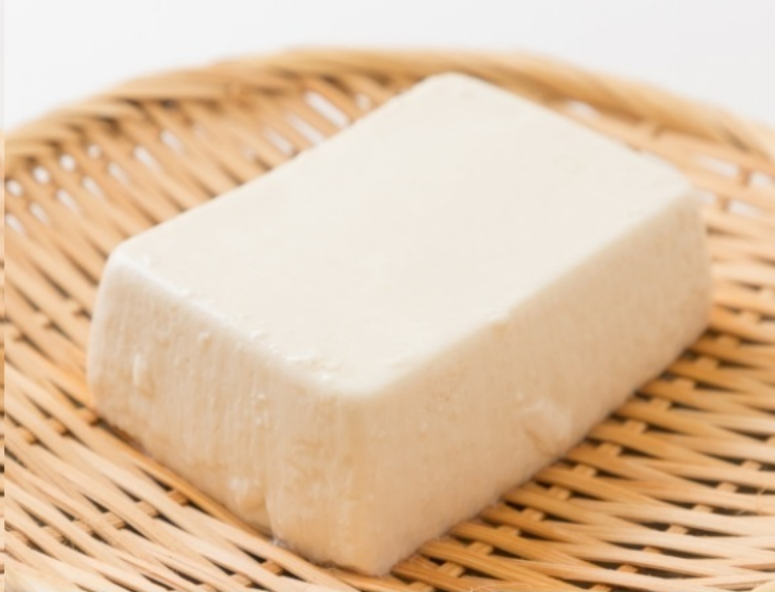

Tofu  
(organic)

Supplement: Supplementary file 5 [file Image_4.pdf]
